# Development and validation of interpretable machine learning models for postoperative pneumonia prediction

**DOI:** 10.3389/fpubh.2024.1468504

**Published:** 2024-12-11

**Authors:** Bingbing Xiang, Yiran Liu, Shulan Jiao, Wensheng Zhang, Shun Wang, Mingliang Yi

**Affiliations:** ^1^Department of Anesthesiology, West China Hospital, Sichuan University, Chengdu, China; ^2^Nursing Department, Chengfei Hospital, Chengdu, China; ^3^Department of Anesthesiology, The Second Affiliated Hospital of Kunming Medical University, Kunming, China; ^4^Department of Anesthesiology, Clinical Medical College and The First Affiliated Hospital of Chengdu Medical College, Chengdu, China; ^5^Department of Anesthesiology, Chengdu Fifth People’s Hospital (The Second Clinical Medical College, Affiliated Fifth People’s Hospital of Chengdu University of Traditional Chinese Medicine), Chengdu, China

**Keywords:** postoperative pneumonia, machine learning, prediction model, risk factors, perioperative medicine

## Abstract

**Background:**

Postoperative pneumonia, a prevalent form of hospital-acquired pneumonia, poses significant risks to patients’ prognosis and even their lives. This study aimed to develop and validate a predictive model for postoperative pneumonia in surgical patients using nine machine learning methods.

**Objective:**

Our study aims to develop and validate a predictive model for POP in surgical patients using nine machine learning algorithms. By evaluating the performance differences among these machine learning models, this study aims to assist clinicians in early prediction and diagnosis of POP, providing optimal interventions and treatments.

**Methods:**

Retrospective data from electronic medical records was collected for 264 patients diagnosed with postoperative pneumonia and 264 healthy control surgical patients. Through correlation screening, chi-square tests, and feature importance ranking, 47 variables were narrowed down to 5 potential predictive factors based on the main cohort of 528 patients. Nine machine learning models, including k-nearest neighbors, support vector machine, random forest, decision tree, gradient boosting machine, adaptive boosting, naive bayes, general linear model, and linear discriminant analysis, were developed and validated to predict postoperative pneumonia. Model performance was evaluated using the area under the receiver operating curve, sensitivity, specificity, accuracy, precision, recall, and F1 score. A distribution plot of feature importance and feature interaction was obtained to interpret the machine learning models.

**Results:**

Among 17,190 surgical patients, 264 (1.54%) experienced postoperative pneumonia, which resulted in adverse outcomes such as prolonged hospital stay, increased ICU admission rates, and mortality. We successfully established nine machine learning models for predicting postoperative pneumonia in surgical patients, with the general linear model demonstrating the best overall performance. The AUC of the general linear model on the testing set was 0.877, with an accuracy of 0.82, specificity of 0.89, sensitivity of 0.74, precision of 0.88, and F1 score of 0.80. Our study revealed that the duration of bed rest, unplanned re-operation, end-tidal CO2, postoperative albumin, and chest X-ray film were significant predictors of postoperative pneumonia.

**Conclusion:**

Our study firstly demonstrated that the general linear model based on 5 common variables might predict postoperative pneumonia in the general surgical population.

## Introduction

Currently, postoperative pneumonia (POP) is the most prevalent hospital-acquired pneumonia worldwide, accounting for approximately 50% of all nosocomial pneumonias (1). It is the third most common complication in all surgical procedures, with an incidence ranging from 1.5 to 15.8% (1–3). Postoperative pneumonia can cause significant harm to surgical patients’ prognosis, even jeopardizing their lives. Reported mortality rates associated with POP in surgical patients range from 9 to 50%, with significant variations depending on the type of surgery, and even after risk adjustment, the 5-year survival rate for patients decreases by 66% (1, 2, 4). Furthermore, POP leads to a significant prolongation of hospital stay, a notable increase in postoperative ICU admission rate, readmission rate, reoperation rate, and mortality rate, greatly burdening patients and their families with additional medical expenses, which can increase by an average of 2 to 10 times (1–5).Early detection of POP is crucial for timely intervention to prevent complications. However, predicting POP has remained challenging (6).

With the rise of 21st-century computer technology and the advent of the big data era, machine learning (ML) have garnered increasing attention in the medical field (7). Research evidence indicates that ML is extensively employed in medicine to establish algorithms and models that effectively classify patients, thereby providing more accurate predictions and diagnoses for diseases, as well as identifying risk factors (8). Given the adverse outcomes, high mortality rates, and substantial healthcare resource consumption caused by postoperative pneumonia globally, further development and validation of predictive models for POP are clearly warranted.

Our study aims to develop and validate a predictive model for POP in surgical patients using nine machine learning algorithms, including K-Nearest Neighbors Classifier (KNN), Support Vector Machine-Radial Basis (SVM-Radial), Random Forest Classifier (RF), Decision Tree Classifier-RPART (RPART), Light Gradient Boosting Machine (GBM), Adaptive Boosting (ADA), Naive Bayes (NB), General Linear Model (GLM), and Linear Discriminant Analysis (LDA). By evaluating the performance differences among these machine learning models, this study aims to assist clinicians in early prediction and diagnosis of POP, providing optimal interventions and treatments. Additionally, it aims to establish a practical foundation for the application of machine learning algorithms in the field of anesthesiology and perioperative medicine.

## Methods

### Human subjects and study design

The study protocol was approved by the Ethics Committee of the Second Affiliated Hospital of Kunming Medical University (No. PJ202139). As this study design is retrospective, the ethics committee waived the requirement for informed consent and clinical trial registration. This study adhered to the applicable TRIPOD guidelines. The data were derived from 17,190 hospitalized patients who underwent surgical treatment at the Second Affiliated Hospital of Kunming Medical University in 2021. In the electronic patient record (EPR) systems of our hospital, a database platform was established by extracting medical records from hospital information system (HIS), anesthesia information management system (AIMS), nosocomial infections surveillance system (NISS), laboratory information system (LIS), picture archiving and communication system (PACS).

The inclusion criteria were used: (1) age over 18 years old; (2) newly developed pneumonia within 30 days after surgery. The patients with the following conditions were excluded from this study: (1) pre-existing pneumonia before operation; (2) tracheal intubation or tracheostomy before operation; (3) procedures outside an operating room; (4) outpatient procedures (hospital stay <24 h); (5) incomplete medical records. Postoperative pneumonia was defined on the basis of US Centers for Disease Control definition of pneumonia (9). Based on the inclusion and exclusion criteria, a total of 264 postoperative pneumonia diagnosed patients and 264 healthy control surgical patients during the same period were included to collect relevant information. Controls were matched by surgical specialty and randomly selected at 1:1 from the remaining surgical patients without pneumonia.

### Variable selection

Based on the review of previous literature and expert recommendations, combined with the actual situation of our hospital, a total of 47 variables were collected through the database platform of the electronic medical record of our hospital: (1) Demographic characteristics: gender, height, weight, gender, and body mass index; (2) Patients’ past medical history: history of smoking, alcohol abuse, hypertension, diabetes, cancer, chronic obstructive pulmonary disease, heart failure, coronary heart disease, stroke, intracerebral hemorrhage, hepatitis, coma and infection; (3) Imaging and laboratory indicators (reviewed as last values before operation or first values after operation): chest X-ray film (mottled or vitreous shadows), pulmonary function test, serum albumin, hemoglobin, blood urea, nitrogen, and creatinine; (4) Surgery-related factors: surgical site, surgical difficulty classification, patient’s operative position (supine or prone), type of surgery (scheduled or emergency), surgery period (day or night), duration of surgery, unplanned re-operation, blood loss, duration of bed rest (duration until patients’ first off-bed activity), and preoperative prophylactic antimicrobial use; (5) Anesthesia-related factors: anesthesia method, ASA physical status, total volume of infusion, red blood cells transfusions, human albumin infusion, duration of mechanical ventilation, mean airway pressure, end tidal CO2, and vasoactive drugs (long-term infusion); (6) Invasive procedures: deep vein catheterization, radial artery cannulation, gastric tube insertion, and indwelling urinary catheter; (7) Outcomes: ICU admission, length of hospital stay, mortality.

### Statistical analysis

All collected raw data was entered into a pre-designed Excel spreadsheet, and statistical analysis were performed using SPSS 23.0. Continuous variables were expressed as mean along with standard deviation or median along with interquartile range. Independent sample t-tests were used for normally distributed data, while Mann–Whitney U tests were used for non-normally distributed data. Categorical variables were expressed as numbers and percentages, and tested using Chi-square tests or Fisher’s exact test.

### Data preprocessing

The original variables contain various data types, therefore, before conducting machine learning, the variables needed to be numerized and categorized, and the variables were analyzed to detect missing values and outliers. In our study, no variable had a missing percentage higher than 1%. For quantitative data, missing values were imputed using the median by group, and for qualitative data, missing values were imputed using the mode by group. The allocation of training and testing sets was randomly divided in a 3:1 ratio using the createDataPartition function in the Rstudio statistical software (R 4.2.2) with the caret package. A predictive model was built based on the training set and externally validated using the testing set.

### Feature selection

Since multicollinearity and confounding variables can affect the performance of the model, various methods were used to select variable features to prevent overfitting and enhance applicability (6). (1) Feature selection based on correlation: Calculate the correlation between features and POP, obtain the indices of feature columns with correlation greater than 0.1, and obtain the corresponding feature names. (2) Feature selection based on statistical analysis: For all categorical data features, calculate the chi-square test for two groups of samples separately, and obtain features with *p* < 0.05. (3) Feature selection based on feature importance: Use a random forest model to obtain feature importance, where a higher MeanDecreaseGini value indicates a higher importance of the feature in the model. Obtain the top 5 features with the highest MeanDecreaseGini. Take the intersection of the above three selection methods and output a Venn diagram to obtain the optimal predictive features.

### Development of the ML model

The construction and validation of the model primarily rely on the “caret” package and “pROC” package in R. A total of nine machine learning classifiers are selected to initially train the training set data. The trainControl function is used to specify training parameters. To build more superior and stable training models, the tune_model function is employed for automatic parameter tuning. This study utilizes a 10-fold cross-validation method to evaluate the generalization performance of the models. This involves dividing the dataset into 10 equal parts, using 9 parts for model training and the remaining 1 part as the validation set to assess model performance and prevent overfitting. These models are evaluated using the receiver operating characteristic curve (ROC) and the area under the receiver operating characteristic curve (AUC) on the test set. The best-performing predictive model is ultimately selected, and the distribution of feature importance is obtained. The interaction between these features is explored and visualized using the “interaction” package in R.

### Performance assessment of the ML model

The Confusion Matrix is the most basic, intuitive, and computationally simple method for measuring the performance of classification models. It provides a more intuitive observation of the accuracy of different classifications, particularly in cases where there is an imbalance in the sample distribution. For binary classification models, the Confusion Matrix is represented by a 2×2 matrix. Our study adopts a 10-fold cross-validation to evaluate the generalization performance of the model. The probability at which the Youden Index is maximized is used as the threshold for classification. The AUC is the main indicator for evaluating the prediction performance of various classification models. Sensitivity, specificity, positive predictive value (PPV), negative predictive value (NPV), accuracy, recall, F1 score, precision, prevalence, balanced accuracy, and threshold are auxiliary evaluation indicators used for performance comparison among different models.

## Results

### Patient characteristics

**Figure 1:**
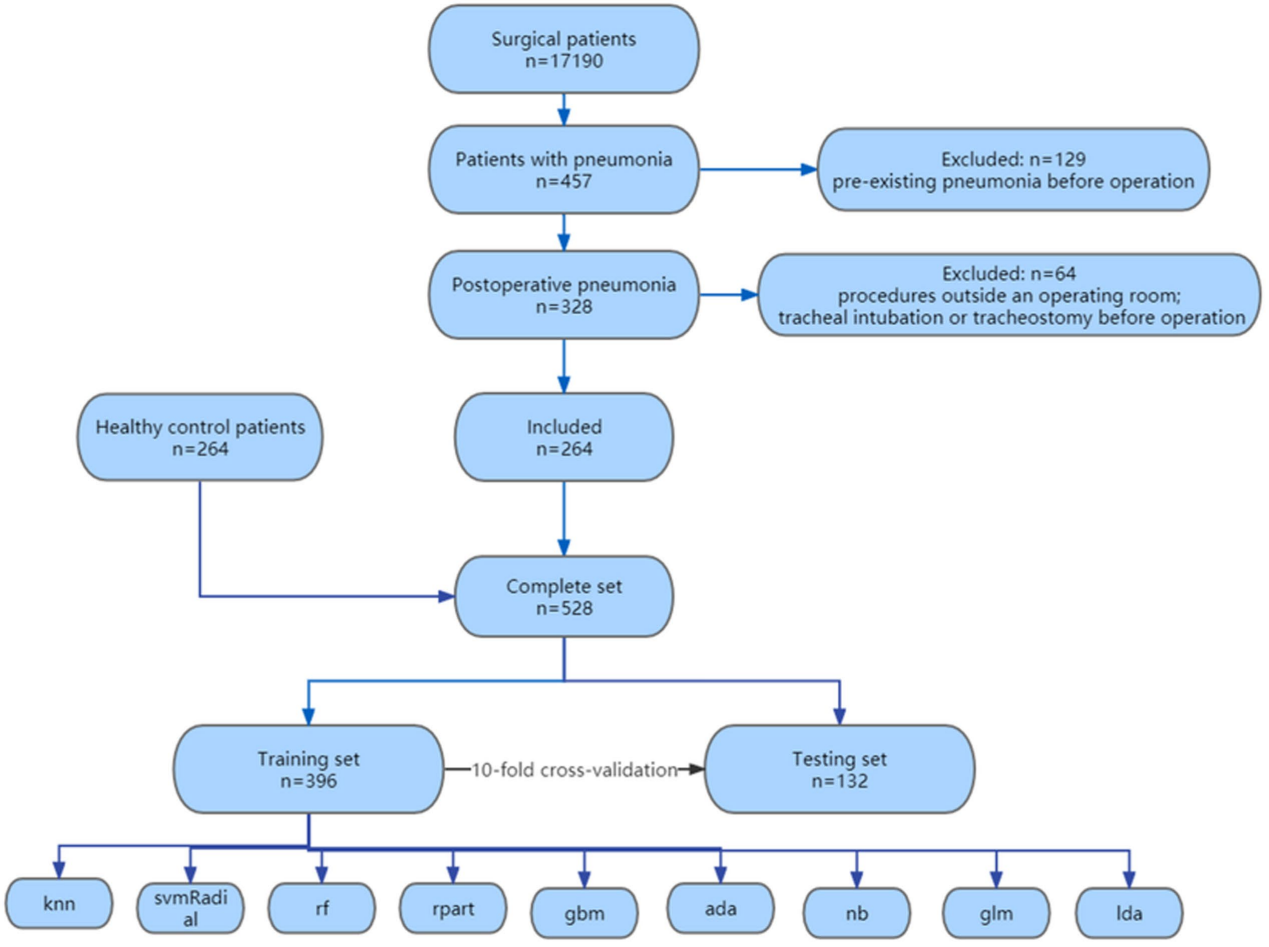
Flow chart of patient enrollment in this study.

A total of 17,190 surgical patients were selected for the study, of which 457 patients were diagnosed with pneumonia. We excluded 129 patient for pre-existing pneumonia before operation, 52 patients for procedures outside an operating room and 12 patients for tracheal intubation or tracheostomy before operation. 264 patients with postoperative pneumonia were included, along with 264 randomly selected healthy control surgical patients during the same period, resulting in a total of 528 patients finally enrolled in our study. The flowchart of the enrollment was shown in Figure 1. The demographic characteristics, laboratory test results, and clinical features of the enrolled patients are shown in Table 1.

**Table 1:** Preoperative characteristics of the study patients.

Variables	Patients with pneumonia (*n* = 264)	Patients without pneumonia (*n* = 264)	*p*_value
Gender			
Male	155(58.71%)	132(50.0%)	0.04
Female	109(41.29%)	132(50.0%)	
Weight (kg)	61.12 ± 10.88	60.35 ± 10.25	0.41
Age (yr)	54.70 ± 14.85	48.73 ± 14.86	<0.001
Body Mass Index			
≥24 kg/m^2^	103(46.8%)	92(35.4%)	0.01
<24 kg/m^2^	117(53.2%)	168(64.6%)	
Hypertension	81(30.7%)	52(19.7%)	0.004
Diabetes mellitus	28(10.6%)	14(5.3%)	0.02
Malignancy	86(32.6%)	52(19.7%)	0.001
Chronic obstructive pulmonary disease	33(12.5%)	6(2.3%)	<0.001
Coronary heart disease	7(2.7%)	6(2.3%)	0.78
Stroke	15(5.7%)	8(3.0%)	0.14
Intracerebral hemorrhage	31(11.7%)	14(5.3%)	0.008
Heart failure	3(1.1%)	0(0%)	0.08
Hypohepatia	7(2.7%)	10(3.8%)	0.46
Renal dysfunction	6(2.3%)	2(0.8%)	0.15
Hepatitis	6(2.3%)	12(4.5%)	0.15
Infection of other sites	23(8.7%)	12(4.5%)	0.05
Chest X-ray film			
Abnormal	136(51.5%)	85(32.2%)	<0.001
Normal	128(48.5%)	179(67.8%)	
Pulmonary function test			
Abnormal	27(10.2%)	8(3.0%)	<0.001
Normal	237(89.8%)	256(97.0%)	
Coma (GCS<8)	34(12.9%)	5(1.9%)	<0.001
History of smoking	112(42.4%)	83(31.4%)	0.009
Alcohol abuse	91(34.5%)	66(25.0%)	0.02
Emergency surgery	58(22.1%)	19(7.2%)	<0.001
Preoperative hemoglobin			
<100 g/L	19(7.2%)	15(5.7%)	0.48
≥100 g/L	245(92.8%)	249(94.3%)	
Postoperative hemoglobin			
<100 g/L	87(33.0%)	44(16.7%)	<0.001
≥100 g/L	177(67.0%)	220(83.3%)	
Preoperative albumin			
<35 g/L	47(17.8%)	40(15.2%)	0.41
≥35 g/L	217(82.2%)	224(84.8%)	
Postoperative albumin			
<35 g/L	198(75.0%)	117(44.3%)	<0.001
≥35 g/L	66(25.0%)	147(55.7%)	
ASA physical status			
≥Grade III	143(54.2%)	106(40.2%)	0.001
<Grade III	121(45.8%)	158(59.8%)	
Anesthesia method			
General anesthesia	258(97.7)	252(95.5%)	0.15
Others	6(2.3)	12(4.5%)	
Duration of ventilation			
≥24 h	81(30.7%)	16(6.1%)	<0.001
<24 h	183(69.3%)	248(93.9%)	
Mean airway pressure			
≥20cmH2O	120(45.5%)	62(23.5%)	<0.001
<20cmH2O	144(54.5%)	202(76.5%)	
ETCO2			
≥40 mmHg	91(34.5)	29(11.0%)	<0.001
<40 mmHg	173(65.5%)	235(89.0%)	
Radial artery cannulation	239(90.5%)	233(88.3%)	0.40
Deep vein catheterization	196(74.2%)	148(56.1%)	<0.001
Total volume of infusion			
≥4,000 mL	104(39.4%)	56(21.2%)	<0.001
<4,000 mL	160(60.6%)	208(78.8%)	
Vasoactive drugs	118(44.7%)	55(20.8%)	<0.001
Red blood cells transfusions	48(18.2%)	19(7.2%)	<0.001
Human albumin infusion	16(6.1%)	21(8.0%)	0.39
Duration of surgery (h)	4.67 ± 3.20	3.40 ± 2.18	<0.001
Intraoperative blood loss (ml)	586.05 ± 1428.03	243.2 ± 256.55	<0.001
Surgical difficulty classification criteria			
≥Grade 4	182(68.9%)	151(57.2%)	0.005
<Grade 3	82(31.1%)	113(42.8%)	
Surgical site			
Thorax or abdomen	140(53.0%)	121(45.8%)	0.10
Other site	124(47.0%)	143(54.2%)	
Operative position			
Prone	17(6.4%)	22(8.3%)	0.41
Supine or lateral	247(93.6%)	242(91.7%)	
Unplanned re-operation	92(34.8%)	7(2.7%)	<0.001
Night operation	66(25.0%)	12(4.5%)	<0.001
Prophylactic antimicrobial use	254 (96.2%)	257 (97.3%)	0.46
Indwelling urinary catheter	209(79.2%)	193(73.1%)	0.10
Gastric tube insertion	84(31.8%)	52(19.7%)	0.001
Duration of bed rest			
≥3 days	177(67.0%)	86(32.6%)	<0.001
<3 days	87(33.0%)	178(67.4%)	
Hospital stay (days)	24.32 ± 14.64	16.16 ± 8.36	<0.001
ICU admission	123(46.6%)	49(18.6%)	<0.001
Mortality	8(3.0%)	0(0%)	0.004

### Univariate analysis

A total of 47 variables were included in this study, 32 of which showed statistical differences in univariate analysis (*p* < 0.05), as follows: gender, age, smoking, drinking, body mass index, hypertension, diabetes, chronic obstructive pulmonary disease, cancer, intracerebral hemorrhage, coma, chest X-ray film, pulmonary function test, surgical difficulty classification, duration of surgery, emergency surgery, night operation, unplanned re-operation, anesthesia method, ASA physical status, duration of ventilation, mean airway pressure, end-tidal CO2, intraoperative blood loss, RBC transfusion, total input, vasoactive agent, deep vein catheterization, gastric tube insertion, postoperative hemoglobin, postoperative albumin, duration of bed rest. These 32 variables were grouped, assigned and encoded as shown in Supplementary Table 1. Compared with the outcome of healthy control group, patients with POP had significantly prolonged hospital stay, increased postoperative ICU admission rate, and increased postoperative mortality rate (*p* < 0.05), as shown in Table 1.

### Feature selection

For ML models such as support vector machines, unlike decision trees or random forests, they do not have the functionality of variable selection. Introducing too many potentially meaningless variables can greatly increase the complexity of the model, hinder algorithm convergence, and may even have a negative impact on the performance of ML models (9, 10). In our study, through correlation screening, chi-square test and feature importance ranking, 47 variables were reduced to 5 potential predictive factors based on the 528 patients in the main cohort. These five features were selected for developing ML models, including chest X-ray film, postoperative albumin, end-tidal CO2, duration of bed rest, and unplanned re-operation.

### Development and performance assessment of the ML model

**Figure 2:**
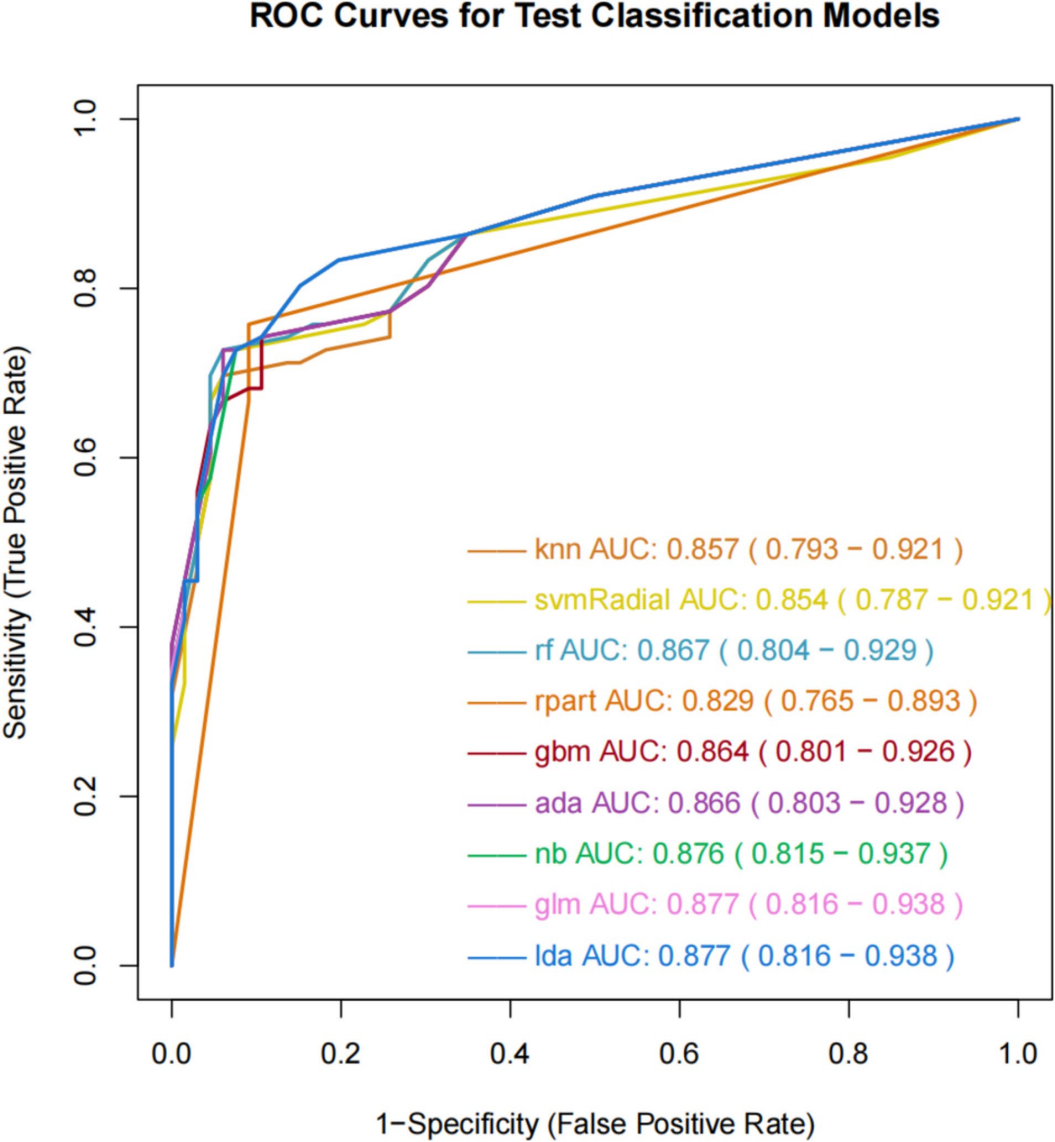
ROC curve and AUC value of machine learning models.

The ROC curve can measure and evaluate the performance of classification models, which are shown in Figure 2. The AUC, representing the area under the ROC curve, is the primary metric used to evaluate classification performance. The AUC typically ranges from 0.5 to 1, with higher values indicating better model performance. The results of the nine ML models on the training set demonstrate similar AUC values ranging from 0.8 to 0.9, indicating good predictive capabilities. GLM and LDA exhibit the highest AUC value of 0.877, followed by NB with 0.876, GBM with 0.864, ADA with 0.866, RF with 0.867, KNN with 0.857, SVM-Radial with 0.854, and RPART with 0.829. Additionally, these nine ML algorithms demonstrate favorable learning curves on the training set, effectively preventing overfitting. GLM and LDA exhibit the best predictive performance, with Youden indices of 0.64.

**Figure 3:**
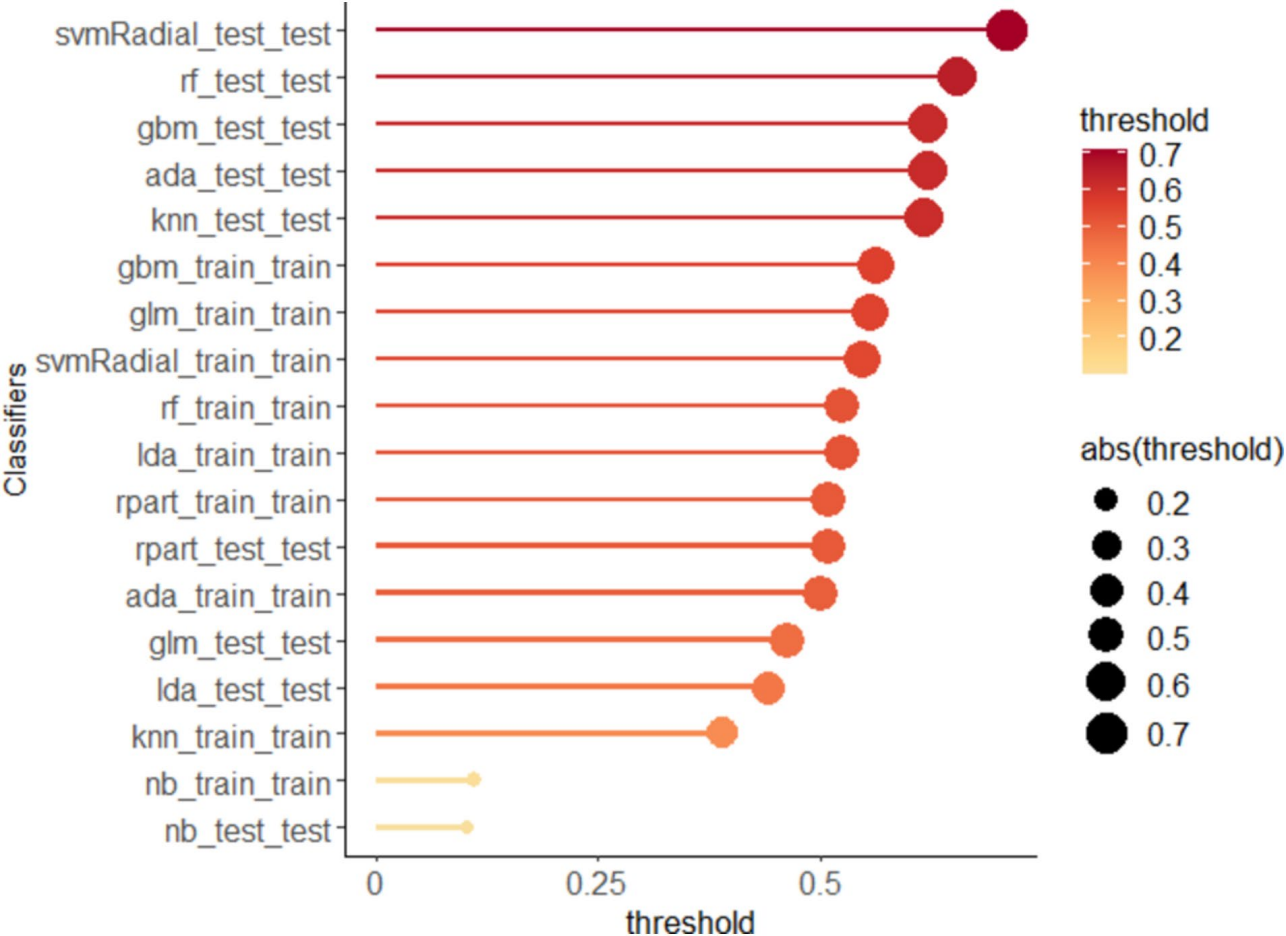
Threshold for all machine learning models.

**Figure 4:**
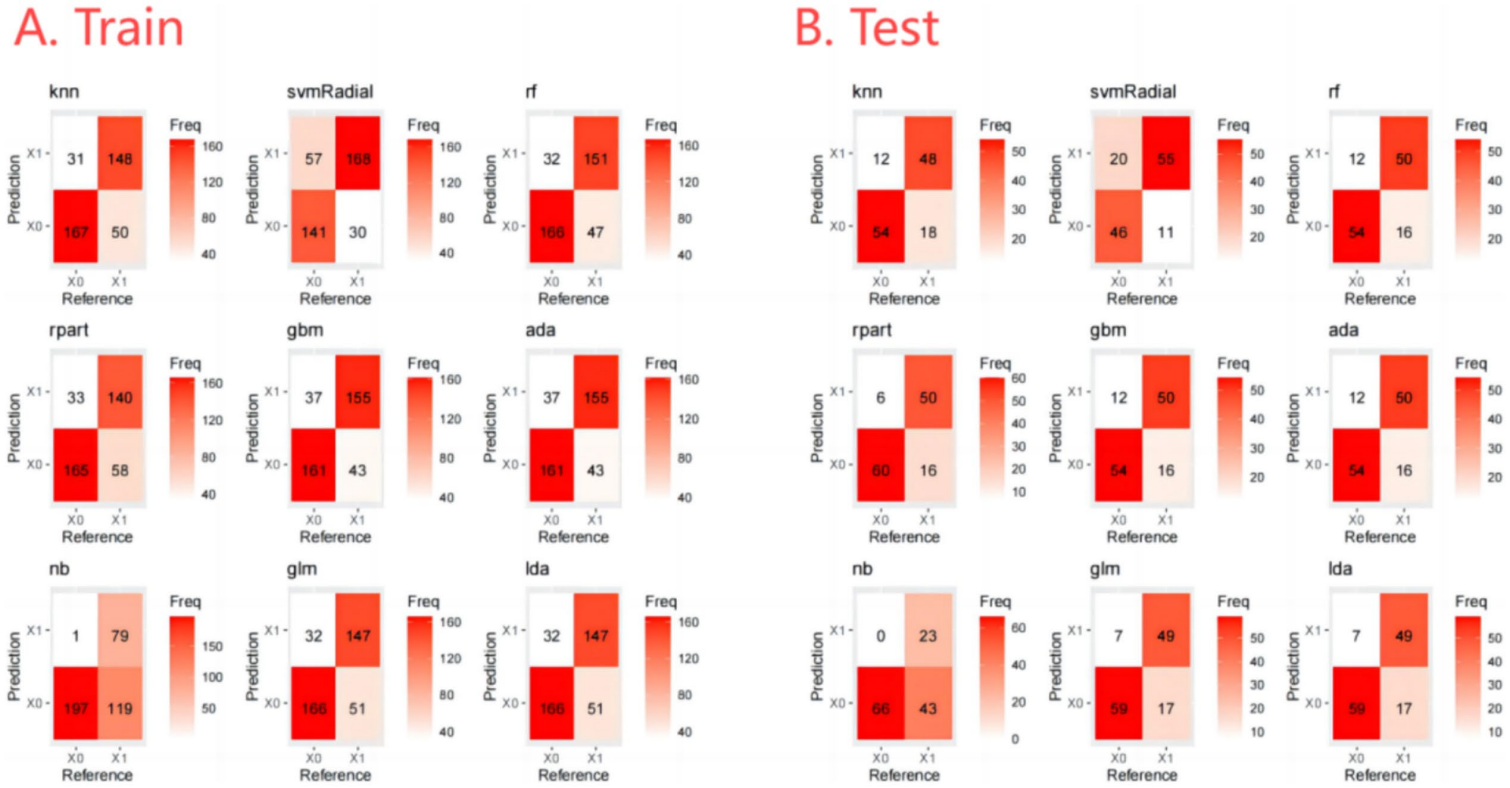
Confusion matrix of training set and testing set.

The output of the classification models includes a probability value ranging from 0 to 1, representing the likelihood of a corresponding sample being predicted as a certain category. A threshold is used to classify the samples, where values greater than the threshold are considered positive and values less than the threshold are considered negative. The optimal thresholds for each ML model are shown in Figure 3. In addition, the Confusion Matrix for the nine ML models, a basic and intuitive visualization tool used in machine learning to showcase model performance, is presented in Figure 4.

**Figure 5:**
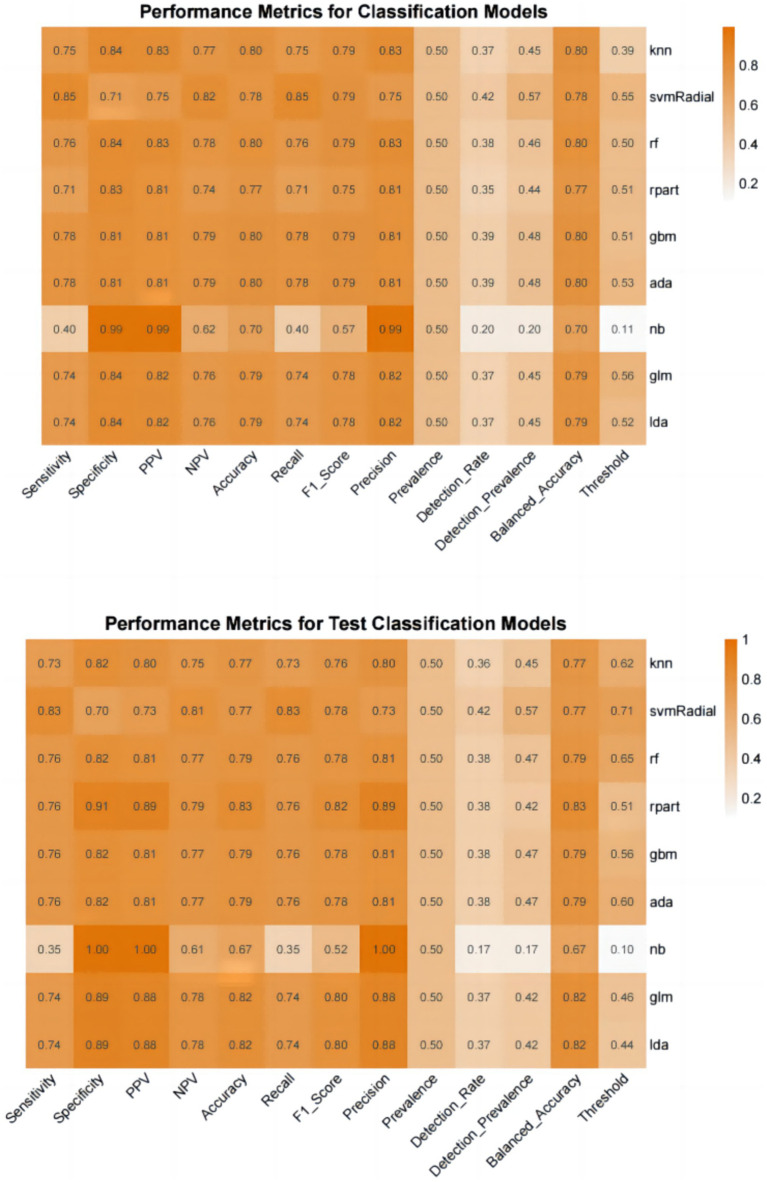
Performance metrics for classification models.

Furthermore, we evaluate the models using multiple auxiliary metrics. Supplementary Table 2 reveals the sensitivity, specificity, accuracy, PPV, NPV, recall, F1 score, precision, prevalence, balanced accuracy, and threshold for all ML models on the training and testing sets. In the training set, GBM achieves the highest accuracy (0.80), NB achieves the highest specificity (0.99), SVM-Radial achieves the highest sensitivity (0.85) and the highest F1 score (0.80). In the testing set, RPART achieves the highest accuracy (0.83), the highest specificity (0.91) and the highest F1 score (0.82), SVM-Radial achieves the highest sensitivity (0.85). Heat maps, a common method for visualizing model performance in machine learning, are generated based on the predictive performance of each model on the training and testing sets, as shown in Figure 5.

**Figure 6:**
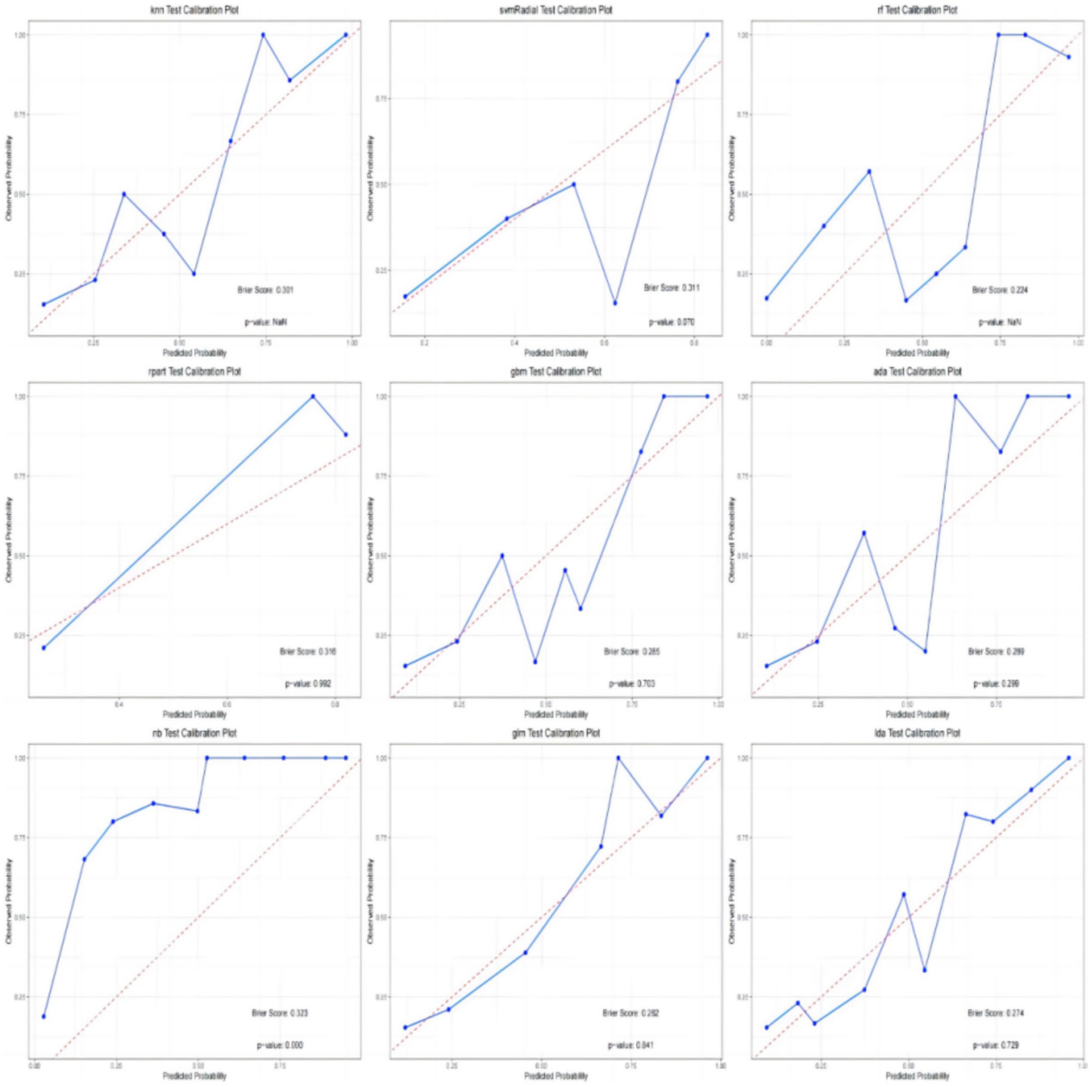
Calibration plot of each model.

**Figure 7:**
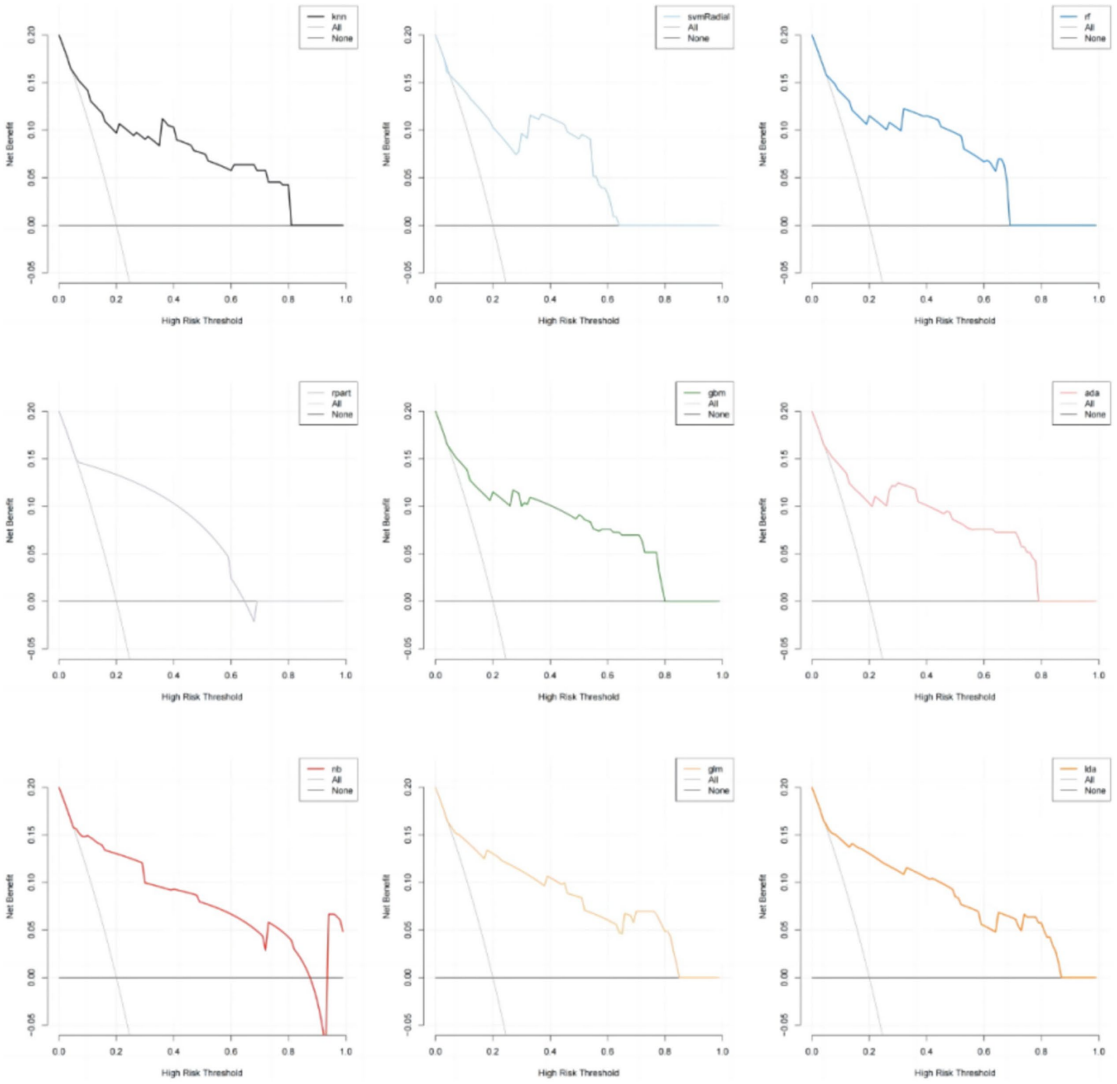
Decision curve analysis.

In addition, the calibration curves and decision curve analysis (DCA) of the validation set for all models are shown in Figures 6, 7. The GLM, LDA and RF models show a modest degree of agreement and fitting between the predicted values and actual values, while other models exhibit poorer agreement. The decision curve analysis of the GLM model suggests that the model yields a positive net benefit in predicting postoperative pneumonia when the probability threshold range is between 0.02 and 0.82.

In conclusion, the results from the training and testing sets indicate that the GLM model, based on five significant features, exhibits the best overall performance. The AUC value for GLM model is 0.877, with a Youden index of 0.64. The GLM model achieves an accuracy of 0.82, an F1 score of 0.80, a specificity of 0.89, and a sensitivity of 0.74. The calibration plot and decision curve analysis shows that the predicted probability of GLM model is in relatively better agreement with the actual probability, and there is a good net benefit in predicting postoperative pneumonia when the threshold probability range is 0.02 ~ 0.82.

### Feature importance

**Figure 8:**
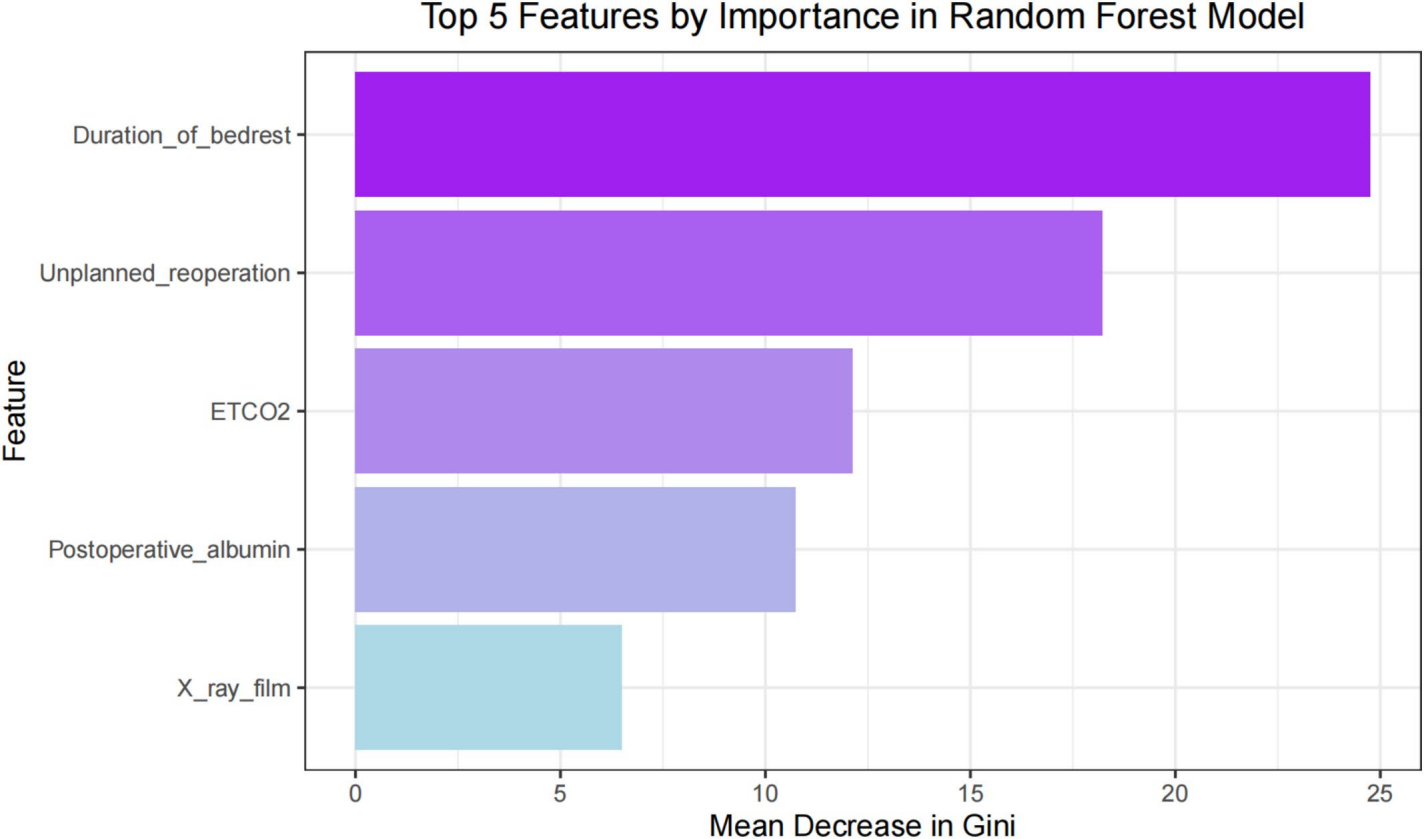
Feature importance.

To ascertain the impact of the five features and their respective attributes included in the predictive model, we obtained a distribution plot of feature importance. This plot illustrates the importance of each feature in correctly predicting positive outcomes (postoperative pneumonia) and negative outcomes (postoperative pneumonia-free), as shown in Figure 8. The features with the highest weights, in descending order, are duration of bed rest, unplanned re-operation, ETCO2, postoperative albumin and chest X-ray film. During the feature selection stage, we also identified other significant features, namely vasoactive agent, age, duration of ventilation, ASA physical status and postoperative hemoglobin. However, due to concerns about introducing too many features and potentially destabilizing the model parameters, only the top five features were included.

**Figure 9:**
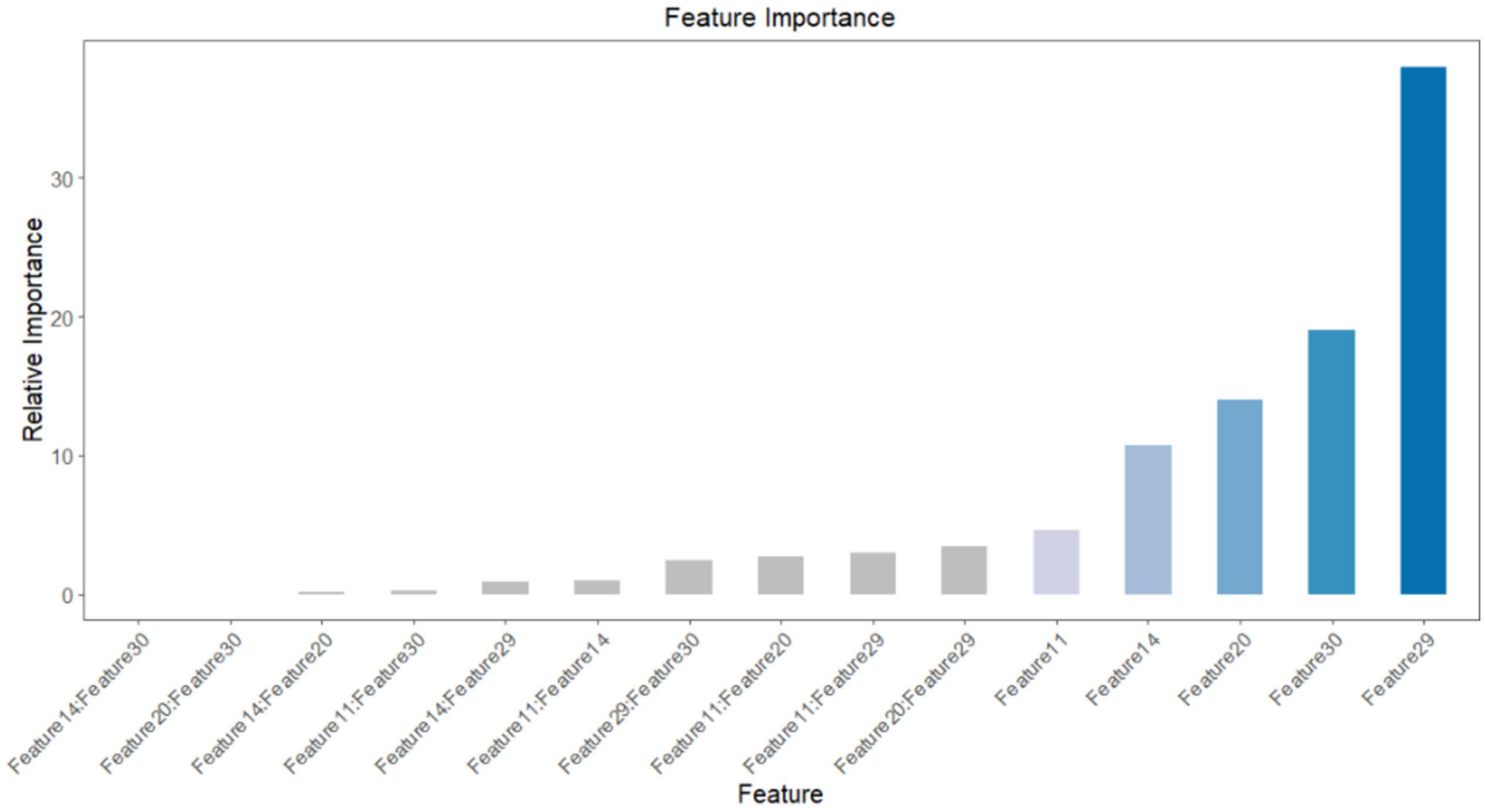
Feature interaction.

In traditional statistical models such as logistic regression, the presence of strong correlations or multicollinearity between independent variables can lead to unstable model parameters, decreased interpretability, reduced predictive performance, and even conclusions that contradict fact. Through interaction analysis, we have shown that there is no strong correlation between these five features, as shown in Figure 9. Therefore, these five features can be used to develop the ML models.

## Discussion

In this study, we successfully established nine machine learning models for predicting postoperative pneumonia in surgical patients. The GLM model demonstrated the best performance, with an AUC value of 0.877. The Youden index for this model was 0.63, with an accuracy of 0.82, specificity of 0.89, sensitivity of 0.74, precision of 0.88, and F1 score of 0.80. It may exhibit even greater predictive and diagnostic advantages in future clinical practice. The calibration plot and decision curve analysis shows that the predicted probability of GLM model is in relatively better agreement with the actual probability, and there is a good net benefit in predicting postoperative pneumonia when the threshold probability range is 0.02 ~ 0.82. To the best of our knowledge, this is the first study utilizing machine learning to predict postoperative pneumonia in the general surgical population.

Currently, POP is still the most common hospital-acquired pneumonia in the world. Our study reports the incidence of postoperative pneumonia is 1.54%, which is closely associated with adverse postoperative outcomes, consistent with previous research findings (2, 3). Thus far, predicting postoperative pneumonia has remained a challenging task, necessitating reliable and accurate predictive models for surgical patients. Considering the global prevalence of antibiotic-resistant bacteria due to antibiotic misuse (8), high specificity is particularly crucial in clinical practice. Therefore, the ML predictive models for postoperative pneumonia can identify high-risk patients promptly, enabling targeted antibiotic prophylaxis for those at high risk of pneumonia during the perioperative period (10). Simultaneously, considering the adverse outcomes and high mortality rates caused by postoperative pneumonia, using ML models to identify high-risk patients and actively formulating appropriate preventive strategies is evidently worthwhile. Therefore, the ML models established in this study can assist clinicians in optimal interventions and treatment, ultimately improving the prognosis of affected patients.

At present, a large number of studies on the risk factors of postoperative pneumonia have been reported. However, these studies have shown significant discrepancies in their findings, limiting the generalizability of their conclusions. Given the substantial variations in risk factors reported in different literature, we believe this further highlights the advantages of ML models in capturing previously unknown correlations within big data (6). Despite the unclear underlying mechanisms, the high clinical relevance of these factors provides a solid foundation for subsequent machine learning processes, enhancing the practicality and clinical value of the conclusions (11, 12). Throughout the perioperative management process, all features involved in the GLM model can be promptly collected, enabling surgeons and anesthesiologists to intervene early during the perioperative period. Additionally, we discovered that factors such as duration of bed rest, unplanned re-operation, ETCO2, postoperative albumin, chest X-ray film, vasoactive agent, age, duration of ventilation, ASA physical status, and postoperative hemoglobin were closely associated with postoperative pneumonia. These findings hold significant clinical implications for perioperative management and contribute to improving clinical outcomes.

In this study, ML models predict postoperative pneumonia in surgical patients through multiple features, avoiding the subjectivity of relying solely on single criteria. Instead, a more comprehensive and objective evaluation of the likelihood of postoperative pneumonia in surgical patients is achieved. For feature selection, random forest can be employed to assess the importance of each feature. The principle behind random forest is based on the concept of ensemble learning (13). By examining the usage of features in different decision trees, the importance score of each feature can be calculated. A higher score indicates a greater impact of the feature on the prediction outcome. The advantage of feature selection based on random forest lies in its ability to handle high-dimensional and sparse features, thereby circumventing the curse of dimensionality associated with traditional feature selection methods (14). Based on random forest, this study identified five features widely used and routinely recorded in clinical practice: chest X-ray film, postoperative albumin, ETCO2, duration of bed rest, and unplanned re-operation. These five features used in constructing the ML model are routinely recorded and widely employed, without requiring any special instruments or devices for acquisition. This indicates the feasibility of our model and its potential for widespread utilization in hospitals. In fact, we aim to utilize this model to calculate the predicted probability of postoperative pneumonia throughout the whole perioperative period. If the prediction result indicates a positive occurrence of postoperative pneumonia, doctors can combine their clinical experience and focus on high-risk patients, promptly providing antibiotic prophylaxis and treatment. This serves as an early warning signal for pneumonia after surgery, prompting surgeons to closely monitor these patients. For instance, it is recommended to conduct temperature checks and blood cultures, make early diagnoses, and select appropriate treatment strategies.

Certainly, the selection of an appropriate ML algorithm is a crucial step, as it depends on the utilized data and desired outcomes (11). It is also important to acknowledge that a single ML algorithm may not capture the interactions among multiple predictors. Therefore, predictive studies may require the comparison of multiple ML algorithms to identify the optimal model (15). In this study, the GLM model demonstrated the best overall performance in predicting postoperative pneumonia, with a maximum AUC value of 0.877, an accuracy of 0.82, an F1 score of 0.80, and a relatively balanced specificity and sensitivity.

In our study, due to the limitations of data set, we finally selected only five features out of the 47 available features in the study, because too many features will affect the stability of the model, which could lead to loss of some important clinical information when using the models for prediction. Of course, since the sample size to develop the prediction model is relatively small, and the performance was evaluated using interval test set, whether the improvement in AUC is clinically significant should be further examined.

## Conclusion

We have successfully developed and validated nine machine learning models for predicting postoperative pneumonia. Among them, the GLM model demonstrated the best overall performance, making it a promising tool for predicting postoperative pneumonia in clinical applications. To the best of our knowledge, this is the first study utilizing machine learning to predict postoperative pneumonia in the general surgical population, offering a novel approach for perioperative management. In the future, a multi-center database containing a large number of medical data is expected to be successfully established and machine learning prediction models for a variety of postoperative complications, including postoperative pneumonia, is looking forward to be constructed.

## Data Availability

The raw data supporting the conclusions of this article will be made available by the authors, without undue reservation.

## Glossary

ML: machine learning
SM: statistical models
POP: postoperative pneumonia
ICU: intensive care unit
ROC: the receiver operating characteristic curve
AUC: the area under the receiver operating characteristic curve
KNN: K-Nearest Neighbors Classifier
SVM-Radial: support vector machine - radial basis
RF: random forest classifier
RPART: decision tree classifier - RPART
GBM: light gradient boosting machine
ADA: adaptive boosting
NB: naive bayes
GLM: general linear model
LDA: linear discriminant analysis
EPR: electronic patient record
HIS: hospital information system
AIMS: anesthesia information management system
NISS: nosocomial Infections surveillance system
LIS: laboratory information system
PACS: picture archiving and communication system
PPV: positive predictive value
NPV: negative predictive value
DCA: decision curve analysis
EHR: electronic health records
EMR: electronic medical records

## References

[1] Xiang B Jiao S Si Y Yao Y Yuan F Chen R. Risk factors for postoperative pneumonia: a case-control study. Front. Public Health. (2022) 10:913897. doi: 10.3389/fpubh.2022.913897, PMID: 35875004 PMC9304902

[2] Wakeam E Hyder JA Tsai TC Lipsitz SR Orgill DP Finlayson SR. Complication timing and association with mortality in the American College of Surgeons' National Surgical Quality Improvement Program database. J Surg Res. (2015) 193:77–87. doi: 10.1016/j.jss.2014.08.025 25260955

[3] Redelmeier DA McAlister FA Kandel CE Lu H Daneman N. Postoperative pneumonia in elderly patients receiving acid suppressants: a retrospective cohort analysis. BMJ. (2010) 340:c2608. doi: 10.1136/bmj.c2608 20566596

[4] Sabaté S Mazo V Canet J. Predicting postoperative pulmonary complications: implications for outcomes and costs. Curr Opin Anaesthesiol. (2014) 27:201–9. doi: 10.1097/ACO.0000000000000045 24419159

[5] Russotto V Sabaté S Canet J. PERISCOPE group; of the European Society of Anaesthesiology (ESA) clinical trial network. Development of a prediction model for postoperative pneumonia: a multicentre prospective observational study. Eur J Anaesthesiol. (2019) 36:93–104. doi: 10.1097/EJA.0000000000000921 30431500

[6] Chen C Yang D Gao S Zhang Y Chen L Wang B. Development and performance assessment of novel machine learning models to predict pneumonia after liver transplantation. Respir Res. (2021) 22:94. doi: 10.1186/s12931-021-01690-3, PMID: 33789673 PMC8011203

[7] Kang J Rancati T Lee S Oh JH Kerns SL Scott JG. Machine learning and Radiogenomics: lessons learned and future directions. Front. Oncologia. (2018) 8:228. doi: 10.3389/fonc.2018.00228, PMID: 29977864 PMC6021505

[8] Kantidakis G Putter H Lancia C Boer J Braat AE Fiocco M. Survival prediction models since liver transplantation - comparisons between cox models and machine learning techniques. BMC Med Res Methodol. (2020) 20:277. doi: 10.1186/s12874-020-01153-1, PMID: 33198650 PMC7667810

[9] TEF A Fowler AJ Pelosi P Gama de Abreu M Møller AM Canet J. A systematic review and consensus definitions for standardised end-points in perioperative medicine: pulmonary complications. Br J Anaesth. (2018) 120:1066–79. doi: 10.1016/j.bja.2018.02.007, PMID: 29661384

[10] Li F Wang C Liu X Peng Y Jin S. A composite model of wound segmentation based on traditional methods and deep neural networks. Comput Intell Neurosci. (2018) 2018:4149103. doi: 10.1155/2018/4149103 29955227 PMC6000917

[11] Thio QCBS Karhade AV Bindels BJJ Ogink PT Bramer JAM Ferrone ML. Development and internal validation of machine learning algorithms for preoperative survival prediction of extremity metastatic disease. Clin Orthop Relat Res. (2020) 478:322–33. doi: 10.1097/CORR.0000000000000997, PMID: 31651589 PMC7438151

[12] Lee T Hwang EJ Park CM Goo JM. Deep learning-based computer-aided detection system for preoperative chest radiographs to predict postoperative pneumonia. Acad Radiol. (2023) 30:2844–55. doi: 10.1016/j.acra.2023.02.016, PMID: 36931951

[13] Zhou CM Xue Q Wang Y Tong J Ji M Yang JJ. Machine learning to predict the cancer-specific mortality of patients with primary non-metastatic invasive breast cancer. Surg Today. (2021) 51:756–63. doi: 10.1007/s00595-020-02170-9, PMID: 33104877

[14] Speiser JL. A random forest method with feature selection for developing medical prediction models with clustered and longitudinal data. J Biomed Inform. (2021) 117:103763. doi: 10.1016/j.jbi.2021.103763, PMID: 33781921 PMC8131242

[15] Woldaregay AZ Launonen IK Årsand E Albers D Holubová A Hartvigsen G. Toward detecting infection incidence in people with type 1 diabetes using self-recorded data (part 1): a novel framework for a personalized digital infectious disease detection system. J Med Internet Res. (2020) 22:e18911. doi: 10.2196/18911, PMID: 32784178 PMC7450374

