# Supplementary material for: Development and validation of interpretable machine learning models for postoperative pneumonia prediction
Source: Front Public Health. 2024 Dec 11;12:1468504. doi: 10.3389/fpubh.2024.1468504 (PMC11670315; doi:10.3389/fpubh.2024.1468504)
Supplement: Supplementary file 1 [file Table_1.docx]

**Supplementary Table 1** Variables and features comparison table

| Features | Variables | Classification | Assignment |
| --- | --- | --- | --- |
| Feature1 | Age | ≥60yr; ＜60yr | 1; 0 |
| Feature2 | Sex | Male；Female | 1; 0 |
| Feature3 | BMI | ≥24kg/m^2^; ＜24kg/m^2^ | 1; 0 |
| Feature4 | History of smoking | Yes; No | 1; 0 |
| Feature5 | Alcohol abuse | Yes; No | 1; 0 |
| Feature6 | Hypertension | Yes; No | 1; 0 |
| Feature7 | Diabetes | Yes; No | 1; 0 |
| Feature8 | COPD | Yes; No | 1; 0 |
| Feature9 | Cancer | Yes; No | 1; 0 |
| Feature10 | Coma | GCS＜8; GCS≥8 | 1; 0 |
| Feature11 | Chest X-ray film | Abnormal; Normal | 1; 0 |
| Feature12 | Emergency surgery | Yes; No | 1; 0 |
| Feature13 | Postoperative hemoglobin | ＜100g/L; ≥100g/L | 1; 0 |
| Feature14 | Postoperative albumin | ＜35g/L; ≥35g/L | 1; 0 |
| Feature15 | Intracerebral hemorrhage | Yes; No | 1; 0 |
| Feature16 | ASA physical status | ≥Grade III; ＜Grade III | 1; 0 |
| Feature17 | Pulmonary function test | Abnormal; Normal | 1; 0 |
| Feature18 | Duration of ventilation | ≥24h；＜24h | 1; 0 |
| Feature19 | Mean airway pressure | ≥20cmH2O; ＜20cmH2O | 1; 0 |
| Feature20 | ETCO2 | ≥40mmHg; ＜40mmHg | 1; 0 |
| Feature21 | RBC transfusion | Yes; No | 1; 0 |
| Feature22 | Total input | ≥4000mL; ＜4000mL | 1; 0 |
| Feature23 | Vasoactive agent | Yes; No | 1; 0 |
| Feature24 | Anesthesia method | General anesthesia；Others | 1; 0 |
| Feature25 | Duration of surgery | ≥3h; ＜3h | 1; 0 |
| Feature26 | Intraoperative blood loss | ≥400mL；＜400mL | 1; 0 |
| Feature27 | Surgical difficulty classification | Grade 4; Others | 1; 0 |
| Feature28 | Night operation | Yes; No | 1; 0 |
| Feature29 | Duration of bed rest | ≥3 days；＜3 days | 1; 0 |
| Feature30 | Unplanned re-operation | Yes; No | 1; 0 |
| Feature31 | Deep vein catheterization | Yes; No | 1; 0 |
| Feature32 | Gastric tube insertion | Yes; No | 1; 0 |
| Outcome | Postoperative pneumonia | Yes; No | 1; 0 |
